# Long‐Term Impact of Childhood Dental Attendance on Perceived Adult Oral Health: The British Cohort Study

**DOI:** 10.1111/jphd.12661

**Published:** 2025-01-11

**Authors:** Aina Najwa Mohd Khairuddin, Jing Kang, Jennifer E. Gallagher

**Affiliations:** ^1^ Dental Public Health, Centre for Host‐Microbiome Interactions, Faculty of Dentistry, Oral and Craniofacial Sciences King's College London London UK; ^2^ Department of Community Oral Health & Clinical Prevention, Faculty of Dentistry Universiti Malaya Kuala Lumpur Malaysia; ^3^ Oral Clinical Research Unit, Faculty of Dentistry, Oral and Craniofacial Sciences King's College London London UK

**Keywords:** adult, children, cohort studies, dental attendance pattern, dental visits, oral health

## Abstract

**Objective:**

To evaluate the effect of childhood dental attendance pattern on self‐rated oral health in middle adulthood among the British population.

**Methods:**

Data from the 1970 British Cohort Study involving participants born in England, Scotland, and Wales were used. Self‐rated oral health was assessed at age 46. Childhood dental attendance patterns, derived from follow‐up surveys at ages 5, 10, and 16, were classified as ‘always’, ‘sometimes’, or ‘never’ regular. Logistic regression was performed to estimate the effect of childhood dental attendance on adult oral health. Subgroup analyses by education level and oral hygiene practices examined potential variations across these factors.

**Results:**

This study analyzed data from 4699 participants. An association was found between childhood dental attendance pattern and self‐rated oral health in middle adulthood. The odds of reporting poor oral health were higher among those with ‘sometimes’ and ‘never’ regular dental attendance patterns than ‘always’ regular attenders. However, this association became insignificant after adjusting for covariates. Subgroup analyses revealed that ‘sometimes’ and ‘never’ regular attendance patterns were associated with higher odds of reporting poor oral health among participants who brushed less than twice daily and those with lower parental academic qualifications.

**Conclusion:**

This study suggests that childhood dental attendance is associated with perceived adult oral health, though this relationship diminished after adjusting for covariates. However, the association persisted among those who brushed less than twice daily and had lower parental academic qualifications. It underscores the importance of both demographic factors and health‐related behaviors in determining long‐term oral health outcomes.

**Abbreviations**
BCS70: 1970 British Cohort Study
CI: Confidence interval
GSROH: Global self‐ratings of oral health
OR: Odds ratio
UK: United Kingdom
VIF: Variance inflation factor

## 1 Background

Oral health is an important component of overall health and significantly impacts an individual's well‐being and quality of life [1]. This highlights the importance of early dental visits, particularly in young populations. In the United Kingdom (UK), the Child Dental Health Survey 2013 reported that 46% of 15‐year‐olds and 34% of 12‐year‐olds had experienced “obvious decay” in their permanent teeth [2]. The Adult Oral Health Survey 2021 further indicated that 19% of adults in England had been diagnosed with gum disease and 25% reported issues with their teeth, fillings, crowns, or fixed bridges [3]. Generally, these oral conditions substantially affect daily functions, loss of school days, reduce study or work efficiency, causing pain and sepsis [4]. Early visits to the dental care provide essential opportunities for promoting good oral hygiene practices, as well as preventive care, such as scaling, fluoride treatments, and dental sealants. Additionally, early detection and management of oral diseases can prevent complications and reduce the need for extensive treatments [5].

While early dental visits and preventive measures are essential for establishing a foundation for lifelong oral health [6, 7, 8], maintaining regular dental visits remains equally important [9]. Annual dental visits among the UK children have been steadily high (89%–90%) from 2003 to 2013 [2]. Visiting trend among the UK adults showed substantial increase in the span of 17 years (1991 to 2008); the proportion rose from 55% to 68% in England, from 48% to 66% in Wales, and from 47% to 67% in Scotland [8]. In Northern Ireland, there was a marginal rise from 63% in 2001 to 66% in 2008. By 2018, the rise in dental service utilization went up to 84% [10]. During the COVID‐19 pandemic, a dramatic decline in dental service utilization was reported when all routine and non‐urgent dental services were ceased during the initial lockdown in March 2020 [11]. This has resulted in longer waiting lists, increased treatment needs and negative impact on individual's quality of life. Although dental activity levels began to rise gradually as lockdown restrictions were eased from June 2020, they have yet to return to pre‐pandemic levels [11].

Despite the United Kingdom being a high‐income country and having large workforce in the general dental services, the prevalence of oral diseases and inequalities in oral health associated with underlying social determinants of health are unsettling [12]. Strategies have been outlined to shape the workforce in support of oral health and deliver contemporary services across the life course [13]. However, the pathways to favorable adult oral health are complex, and there are still some gaps in knowledge that need to be addressed. To better understand the influence of dental attendance patterns on oral health over time, the life course approach provides a valuable perspective [14]. It considers the cumulative effects of social, behavioral, and biological factors from early life to older age, offering insights into the long‐term impacts of dental care behaviors.

Evidence from a systematic review involving 11 longitudinal studies from five countries (Sweden, New Zealand, Australia, Hong Kong, and Brazil) underscores the long‐term benefits of regular childhood dental visits on adult oral health [9]. The review reported that individuals who had consistent dental care in early life reported better oral health‐related quality of life, less dental caries experience, and fewer missing teeth in later years. The findings, however, might not be applicable to other populations, particularly the British population. The UK national surveys suggested that non‐regular dental attenders had significantly higher dental caries experience and numbers of decayed and missing teeth, with fewer filled teeth as opposed to their counterparts, even after adjustment for confounders [15]. While this cross‐sectional study offers insight into the effect of dental attendance on oral health, further research is needed to understand its impact throughout an individual's life course. This study aims to investigate the association between childhood dental attendance and perceived adult oral health. Specifically, we hypothesize that childhood dental attendance is positively associated with perceived oral health in adulthood among the UK population.

## 2 Methods

### 2.1 Study Population

**FIGURE 1:**
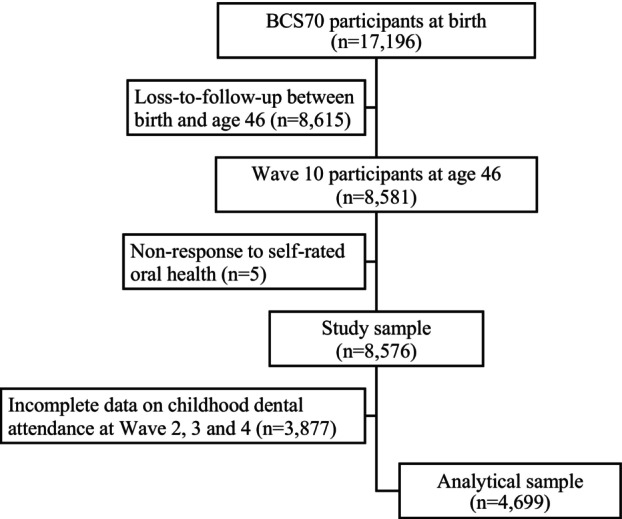
Analytical sample derivation for this study.

This study utilized data from the 1970 British Cohort Study (BCS70) involving more than *n* = 17,000 individuals born in a single week in April 1970 across England, Scotland, and Wales [16]. There have been 11 waves of data collection to date, with information collected from parents at birth and during childhood, whereas the cohort members started to provide information from later childhood [17]. This study focused on Waves 2, 3, 4, and 10, corresponding to the cohort members' ages of 5, 10, 16, and 46 years. Data from all *n* = 8581 participants in Wave 10 were retrieved. Of that, *n* = 8576 provided data for self‐rated oral health. Subsequently, only those that had complete data on dental attendance in Waves 2, 3, and 4 were included in the analysis, which resulted in the final sample of *n* = 4699 participants (see Figure 1).

### 2.2 Measures

The oral health outcome at age 46 was assessed through self‐rated responses (*“Would you say that your dental health (your mouth, teeth, and/or denture) is…”*), categorized on a five‐point scale (excellent, very good, good, fair, poor). Those who responded excellent, very good, or good were considered as having good oral health, while those who rated fair or poor as having poor oral health. The exposure was childhood dental attendance patterns, derived from self‐reported dental visit within the past 12 months at each time point—age 5, 10, and 16 years. These patterns were classified into three categories based on the reported dental visits across the three waves: ‘always’ regular (visited dental personnel at each wave), ‘sometimes’ regular (missed dental visit once), and ‘never’ regular (missed dental visit more than once).

The participants' demographic, behavioral characteristics, and comorbidities were extracted as covariates to assess the association between childhood dental attendance and adult oral health. Covariates during childhood were measured at baseline in Wave 2 (age 5), except for sugary food and beverages intake which were obtained in Wave 3 (age 10) and toothbrushing frequency in Wave 4 (age 16). The childhood demographic variables included sex (male or female), ethnicity (White or others), country (England, Scotland or Wales), parent's academic qualification (high or low), and parent's social class based on Registrar General's Social Class (professional/managerial/technical, skilled nonmanual/manual or partially skilled/unskilled) [18]. Behavioral covariates during childhood involved sugary intake of food and beverages (often or sometimes) [19] and toothbrushing frequency (less than twice daily, at least twice daily or unknown). Additionally, childhood comorbidities, including both physical and mental illnesses (yes or no), were considered. In adulthood, the covariates during adulthood were measured in Wave 10 (age 46). These included social class, determined by the National Statistics Socioeconomic Classification (higher managerial/administrative/professional, intermediate or routine/manual occupations) [20], smoking status (yes or no), and comorbidities (yes or no).

### 2.3 Data Analysis

**FIGURE 2:**
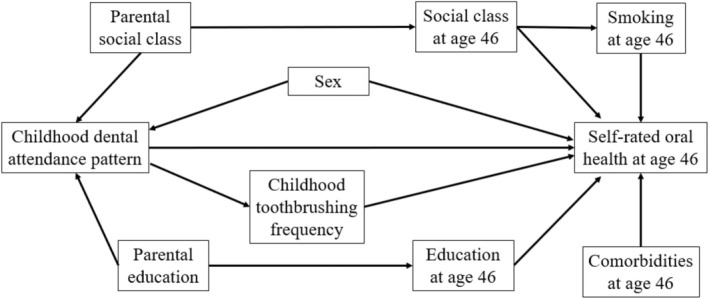
Direct acyclic graph for self‐rated oral health at age 46. The potential pathways through which childhood dental attendance affects self‐perceived oral health in later life include sociodemographic factors and oral health‐related behaviors.

This study used directed acyclic graph (DAG) to illustrate the underlying association between childhood dental attendance pattern and perceived adult oral health, while considering oral health‐related covariates that might influence the association. Assumptions about the causal pathways are illustrated in Figure 2. In summary, parental sociodemographic factors were assumed to have indirect effects on perceived adult oral health through adult sociodemographic factors [21, 22, 23]. Sex [24], toothbrushing frequency [25, 26], smoking habit [27], and comorbidity [24] were assumed to have direct effects on perceived adult oral health.

All analyses were carried out using Stata 17.0 [28]. The participants' characteristics were stratified by self‐rated oral health and compared using the Chi‐squared test. Missing data were assumed to be missing at random and handled by using multiple imputation to restore sample representativeness, increase power, and reduce bias [29]. Multiple imputation with 20 iterations was performed on covariates with missing data. The covariates, exposure, and outcome variables with complete data were included in the imputation equation to assist in the estimation of missing values for variables with missing data. By including these variables, the imputation model can better capture the underlying structure of the data and produce more reliable imputed values [29]. The correlation between dental attendance pattern and other covariates was tested to ascertain whether there is presence of multicollinearity in the regression model. Variance inflation factors (VIFs) were calculated to measure the extent of collinearity between predictors. Multicollinearity was said to exist if the VIF values were greater than 5, and this was addressed by removing or combining the highly correlated variables [30]. Logistic regression was performed to estimate the association between dental attendance patterns and self‐rated oral health in crude and adjusted models. Subgroup analyses by academic qualification and toothbrushing frequency were also conducted to uncover hidden patterns that were overlooked in the overall analysis. The results were reported in odds ratios (ORs) and 95% confidence intervals (CIs). The significance level (*p* value) was set at 0.05. Sensitivity analysis was conducted to compare results between the imputed and complete case data sets.

## 3 Results

This study analyzed *n* = 8576 cohort members of the BCS70. Table 1 shows the characteristics of the participants, whereby a quarter of them (25%) reported poor oral health. The proportion of people who reported having poor oral health was significantly higher among males, those who were not in professional/managerial class and had low academic qualification during childhood and adulthood, those who did not brush teeth at least twice daily and had higher sugary food intake during childhood, those with comorbidities during childhood and adulthood, as well as smokers. Bivariate analysis in Table 2 reveals that those who ‘always’ attended dental visits regularly during childhood tend to rate their oral health as good, while those who ‘sometimes’ or ‘never’ attended regularly reported poor oral health more frequently in middle adulthood.

**TABLE 1:** Self‐rated oral health at age 46 by sociodemographic and oral health‐related characteristics.

Characteristics	Self‐rated oral health		
	Good	Poor	*p*
	*n* (%)	*n* (%)	
Total, *n* = 8576	6433 (75.01)	2143 (24.99)	
Sex			**< 0.001**
Male	2926 (45.48)	1225 (57.16)	
Female	3507 (54.52)	918 (42.84)	
Ethnicity			0.541
White British	5551 (95.51)	1836 (95.87)	
Others	261 (4.49)	79 (4.13)	
Country			0.933
England	5584 (86.92)	1858 (86.98)	
Wales	325 (5.06)	111 (5.20)	
Scotland	515 (8.02)	167 (7.82)	
Parent's social class			**< 0.001**
Professional/managerial/technical	2036 (33.19)	475 (23.63)	
Skilled nonmanual/manual	3142 (51.21)	1120 (55.72)	
Partially skilled/unskilled	957 (15.60)	415 (20.65)	
Parent's academic qualification			**< 0.001**
High qualification	1748 (28.72)	433 (21.61)	
Low qualification	4339 (71.28)	1571 (78.39)	
Childhood comorbidities			**< 0.001**
No	2406 (38.23)	708 (33.81)	
Yes	3888 (61.77)	1386 (66.19)	
Childhood sugary food intake			**0.024**
Sometimes	1729 (30.73)	502 (27.89)	
Often	3897 (69.27)	1298 (72.11)	
Childhood sugary drinks intake			0.352
Sometimes	1001 (17.77)	302 (16.78)	
Often	4631 (82.23)	1498 (83.22)	
Childhood toothbrushing frequency			**< 0.001**
≥ twice daily	1900 (37.01)	427 (26.57)	
< twice daily	752 (14.65)	277 (17.24)	
Unknown	2482 (48.34)	903 (56.19)	
Adulthood comorbidities			**< 0.001**
No	2777 (43.17)	709 (33.08)	
Yes	3656 (56.83)	1434 (66.92)	
Adulthood smoking			**< 0.001**
No	5417 (84.21)	1382 (64.49)	
Yes	1016 (15.79)	761 (35.51)	
Adulthood academic qualification			**< 0.001**
High qualification	2740 (43.13)	605 (29.00)	
Low qualification	3613 (56.87)	1481 (71.00)	
Adulthood social class			**< 0.001**
Higher managerial/administrative/ professional occupations	3010 (54.38)	706 (42.02)	
Intermediate occupations	1260 (22.77)	387 (23.04)	
Routine and manual occupations	1265 (22.85)	587 (34.94)	
Adulthood sugary food intake			0.996
No	1574 (33.83)	438 (33.77)	
Yes	3079 (66.17)	859 (66.23)	
Adulthood sugary drinks intake			0.759
No	718 (15.43)	195 (15.03)	
Yes	3935 (84.57)	1102 (84.97)	

*Note:* Missing data out of study sample, *n* = 8576: ethnicity (9.90%), country (0.19%), parent's social class (5.03%), parent's academic qualification (5.66%), childhood sugary food intake (13.41%), childhood sugary drinks intake (13.34%), childhood comorbidity (2.19%), adulthood social class (15.87%), adulthood academic qualification (1.60%), adulthood sugary food intake (30.62%), adulthood sugary drinks intake (30.62%). Bold indicates highlight results with statistical significance.

**TABLE 2:** Self‐rated oral health at age 46 by childhood dental attendance pattern.

Characteristics	Self‐rated oral health		
	Good	Poor	*p*
	*n* (%)	*n* (%)	
Total, *n* = 8576	6433 (75.01)	2143 (24.99)	
Childhood dental attendance pattern			**< 0.001**
Always regular	2306 (63.39)	606 (57.12)	
Sometimes regular	1155 (31.75)	383 (36.10)	
Never regular	177 (4.86)	72 (6.78)	

*Note:* Missing data out of study sample, *n* = 8576: childhood dental attendance pattern (45.21%). Bold indicates highlight results with statistical significance.

In the logistic regression model, *n* = 4699 participants with childhood dental attendance information were included in the analysis. Based on the crude model, the odds of ‘sometimes’ regular attenders rating their oral health as poor were 1.26 times higher (95% CI = 1.09–1.46) than the ‘always’ regular attenders, with even higher odds observed among the ‘never’ regular attenders (crude OR = 1.55, 95% CI = 1.16–2.06). Interestingly however, this association did not hold after adjusting for covariates (‘sometimes’ regular adjusted OR = 1.11, 95% CI = 0.95–1.29; ‘never’ regular adjusted OR = 1.14, 95% CI = 0.84–1.55) (Table 3). Multicollinearity was found to be absent (mean VIF = 1.13), suggesting that the exposure and covariates were not correlated to one another. Additionally, the sensitivity analysis showed similar overall findings, except that the imputed data set produced larger effect with more precise estimates than the complete case data set [see Data S1]. The absence of multicollinearity and the sensitivity analysis with imputed data reinforced the robustness of our findings.

**TABLE 3:** Multivariate logistic regression for self‐rated oral health at age 46 years.

	Crude model		Adjusted model	
	OR [95% CI]	*p*	OR [95% CI]	*p*
	*n* = 4699			
Childhood dental attendance pattern				
Always regular	(Reference)		(Reference)	
Sometimes regular	**1.26 [1.09; 1.46]**	**0.002**	1.11 [0.95; 1.29]	0.190
Never regular	**1.55 [1.16; 2.06]**	**0.003**	1.14 [0.84; 1.55]	0.396
Sex				
Male			(Reference)	
Female			**0.60 [0.52; 0.70]**	**< 0.001**
Parent's social class				
Professional/managerial/technical			(Reference)	
Skilled non‐manual/manual			**1.28 [1.08; 1.52]**	**0.005**
Partially skilled/unskilled			**1.59 [1.27; 1.99]**	**< 0.001**
Childhood sugary food intake				
Sometimes			(Reference)	
Often			1.07 [0.91; 1.25]	0.443
Childhood toothbrushing frequency				
≥ twice daily			(Reference)	
< twice daily			**1.52 [1.24; 1.86]**	**< 0.001**
Unknown			**1.26 [1.07; 1.49]**	**0.005**
Childhood comorbidity				
No			(Reference)	
Yes			1.16 [0.99; 1.37]	0.063
Adulthood social class				
Higher managerial/administrative/ professional			(Reference)	
Intermediate			1.04 [0.85; 1.27]	0.727
Routine/manual			**1.47 [1.23; 1.75]**	**< 0.001**
Smoking				
No			(Reference)	
Yes			**2.60 [2.20; 3.07]**	**< 0.001**
Adulthood comorbidity				
No			(Reference)	
Yes			**1.66 [1.43; 1.93]**	**< 0.001**

*Note:* Bold indicates highlight results with statistical significance.

Abbreviations: CI, confidence interval; OR, odds ratio.

In the subgroup analyses, among those whose parents had low academic qualifications, individuals with ‘sometimes’ regular dental attendance pattern during childhood were more likely to rate their oral health as poor in middle adulthood (crude OR = 1.26, 95% CI = 1.06–1.50; adjusted OR = 1.20, 95% CI = 1.01–1.44) than those ‘always’ attended dentist regularly (Table 4). Additionally, individuals with ‘never’ regular childhood attendance pattern had higher odds of perceiving poor oral health at age 46 than those with ‘always’ regular attendance pattern. This was observed in both high (crude OR = 2.06, 95% CI = 1.05–4.05) and low (crude OR = 1.38, 95% CI = 1.00–1.91) parental academic qualification categories. However, after adjusting for covariates, these differences were no longer significant. When examining childhood toothbrushing frequency (Table 5), we found that among those who brushed less than twice daily, individuals with ‘sometimes’ regular (crude OR = 1.55, 95% CI = 1.12–2.15) and ‘never’ regular (crude OR = 2.32, 95% CI = 1.37–3.94) childhood dental visits had higher odds of reporting poor oral health at age 46 than those with ‘always’ regular dental visits. Additionally, among those who brushed at least twice daily but ‘never’ regularly attended dental visits, there was a higher likelihood of reporting poor oral health than those who ‘always’ attended regularly (crude OR = 1.90, 95% CI = 1.11–3.26). However, after adjusting for covariates, only those who brushed less than twice daily and ‘never’ regularly attended dental visits showed a significant difference in self‐rated oral health (adjusted OR = 2.00, 95% CI = 1.15–3.47).

**TABLE 4:** Logistic regression for self‐rated oral health at age 46 years by parent's academic qualification during childhood.

	High academic qualification				Low academic qualification			
	Crude	*p*	Adjusted		Crude	*p*	Adjusted	*p*
	OR (95% CI)		OR (95% CI)	*p*	OR (95% CI)		OR (95% CI)	
Total, *n* = 4699	*n* = 1509				*n* = 3190			
Childhood dental attendance pattern								
Always regular	Reference		Reference		Reference		Reference	
Sometimes regular	1.09 (0.82–1.47)	0.540	0.99 (0.73–1.35)	0.958	**1.26 (1.06–1.50)**	**0.007**	**1.20 (1.01–1.44)**	**0.040**
Never regular	**2.06 (1.05–4.05)**	**0.036**	1.75 (0.84–3.67)	0.137	**1.38 (1.00–1.91)**	**0.048**	1.18 (0.84–1.66)	0.349

*Note:* Bold indicates highlight results with statistical significance.

Abbreviations: CI, confidence interval; OR, odds ratio.

**TABLE 5:** Logistic regression for self‐rated oral health at age 46 years by childhood toothbrushing frequency.

	At least twice daily				Less than twice daily				Unknown			
	Crude	*p*	Adjusted		Crude	*p*	Adjusted	*p*	Crude	*p*	Adjusted	*p*
	OR (95% CI)		OR (95% CI)	*p*	OR (95% CI)		OR (95% CI)		OR (95% CI)		OR (95% CI)	
Total, *n* = 4699	*n* = 1817				*n* = 819				*n* = 2063			
Childhood dental attendance pattern												
Always regular	Reference		Reference		Reference		Reference		Reference		Reference	
Sometimes regular	1.15 (0.88–1.49)	0.313	1.00 (0.76–1.32)	0.984	**1.55 (1.12–2.15)**	**0.009**	1.35 (0.96–1.91)	0.085	1.17 (0.95–1.45)	0.145	1.08 (0.87–1.36)	0.479
Never regular	**1.90 (1.11–3.26)**	**0.019**	1.53 (0.86–2.72)	0.143	**2.32 (1.37–3.94)**	**0.002**	**2.00 (1.15–3.47)**	**0.014**	0.90 (0.56–1.45)	0.675	0.64 (0.38–1.08)	0.092

*Note:* Bold indicates highlight results with statistical significance.

Abbreviations: CI, confidence interval; OR, odds ratio.

## 4 Discussion

This study suggests that regular dental visits during childhood may have a positive influence on perceived oral health in middle adulthood. However, similar to other studies [31, 32], this effect was attenuated after accounting for various demographic and behavioral factors, indicating that broader determinants of health play a crucial role in shaping long‐term oral health outcomes. This study also discovered that childhood dental visits had a more pronounced impact on self‐rated oral health at middle adulthood (age 46) among individuals whose parents had low academic qualifications and those who brushed their teeth less frequently in childhood. This emphasizes the influence of parental education on children's oral health behaviors [33, 34] and supports previous studies suggesting that childhood toothbrushing frequency interacts with dental attendance patterns [25], thereby influencing long‐term oral health outcomes [26].

A life‐course perspective provides an opportunity to understand oral health over time [35]. Several mechanisms elucidate how childhood experiences influence oral health in adulthood. For example, children who have non‐regular dental attendance or who are raised in disadvantaged socioeconomic circumstances are more likely to engage in smoking and exhibit poor oral hygiene behaviors [22]. These factors, in turn, elevate the risk of experiencing dental caries and other oral conditions [36, 37]. Additionally, people from low socioeconomic status are more likely to experience oral health problems, yet less likely to access oral healthcare services than those from high categories [15]. This disparity is often attributed to barriers such as costs, lack of access to dental services, and lower prioritization of oral health among children from low socioeconomic backgrounds [38, 39]. According to the UK Oral Health Survey, nearly one‐fifth of adults delayed their dental care due to treatment costs, and 42% of them perceived poor oral health status [40]. Conversely, children from higher socioeconomic backgrounds are more likely to attend regular dental visits, benefiting from better access to dental services, higher parental education levels, and greater health literacy [22]. Another important consideration related to our findings is the role of self‐efficacy in shaping oral health behaviors. Research has shown that individuals with higher self‐efficacy are more likely to engage in preventive measures and seek dental care [41, 42]. Conversely, those with low self‐efficacy may feel overwhelmed by the perceived complexity of maintaining oral health, which can lead to neglect of dental care, despite understanding its importance and potential consequences [43, 44]. Additionally, the influence of self‐efficacy extends beyond individual behaviors to affect broader health outcomes. For instance, studies have shown that adults with higher self‐efficacy report better subjective oral health and oral health‐related quality of life [45, 46].

The overall findings of this study highlight the importance of promoting regular dental visits for children as a public health priority. Interventions such as school‐based dental programs, public awareness campaigns, and oral health prevention policies that expand access to dental care can significantly improve oral health outcomes. School‐based dental programs, for example, can provide preventive care and education to children who might otherwise lack access to dental services [47]. Additionally, this study could inform guidelines, such as those from the National Institute for Health and Care Excellence [48], facilitating the development of a framework for expanding preventive care in dental services. It could also support the use and implementation of Delivering Better Oral Health [5], providing a basis for the dental personnel at the primary care level to assign suitable dental recall intervals based on patients' disease levels and risk of dental disease. Early intervention in conditions such as cavities and malocclusion may prevent these problems from escalating into more severe health concerns. For instance, untreated cavities can lead to tooth loss, which negatively impacts chewing function and overall quality of life. Early loss of deciduous teeth can disrupt the alignment of permanent teeth, leading to crowding or malocclusion, which may also negatively affect psychosocial well‐being, impacting confidence and social interactions. Moreover, policies aimed at improving access to dental services, particularly for low‐income families or those in care, are crucial in ensuring that financial barriers and social factors do not prevent them from receiving necessary dental care. Programs like Smile4Life in England, which includes the Starting Well initiatives, are examples of targeted efforts to improve access for vulnerable groups [49]. The Smile4Life promotes good oral health through prevention, while Starting Well specifically targets children under the age of 3 years living in areas with high levels of dental disease. These initiatives raise public and professional awareness to promote regular dental visits, as well as provide additional support, such as dietary advice, fluoride interventions, and support for oral health behavior change. Expanding such initiatives with increasing access to preventive care would help reduce oral health inequalities and promote equitable access to dental services.

Notably, our findings differ from those reported in a systematic review of longitudinal studies that examined the long‐term impact of dental attendance patterns on oral health [9]. These discrepancies may be attributed to several factors, including differences in sample sizes, study populations, and the nature of dental services. For example, our study tracked participants from childhood through adulthood, whereas other studies, despite having comparable sample sizes, focused on participants in elderhood and found a significant association between dental attendance and oral health‐related quality of life [50, 51]. There are studies that observed cohorts from childhood to adulthood and reported significant findings, however, those studies analyzed smaller sample sizes than ours [36, 37]. Additionally, the context of oral healthcare services also plays a critical role in shaping the long‐term oral health outcomes. In the United Kingdom, dental care is provided free of charge for children, while adults receive care on a fee‐for‐service basis [13]. In New Zealand, comprehensive free dental care is offered to children, primarily through the Community Oral Health Service [52], while adults generally bear the cost of their dental care out‐of‐pocket [53]. In contrast, Sweden provides free dental care for children and young people up to the age of 23, while adult dental care partially subsidized through a national insurance system with a high‐cost protection scheme to reduce the financial burden of extensive treatments [54]. These variations highlight the importance of considering contextual factors such as demographic differences and healthcare systems when interpreting the impact of dental attendance patterns on long‐term oral health outcomes.

One of the strengths of this study is its large sample size and the longitudinal design, which allows for the observation of long‐term trends and associations with clear temporal order. The consideration of time‐varying covariates in childhood and adulthood provides a nuanced understanding of the factors influencing oral health. Additionally, the use of multiple imputation techniques to handle missing data enhances the robustness of the findings. For variables with a large proportion of missing data and uncertainty about whether the data are missing at random (e.g., toothbrushing frequency has 61% missing data out of the study sample, *n* = 8576), the missing data were categorized as ‘unknown.’ This was to avoid extensive imputation which could affect the precision of the findings.

Despite the strengths, there are limitations to consider. First, this study used a subjective oral health indicator, which might introduce measurement bias. It is possible that the ‘sometimes’ regular attenders received dental treatment at one of their visits, which led to their perception of having poor oral health or they might undergo restorative or curative treatments rather than preventive treatments. However, this measurement is adopted from the global self‐ratings of oral health (GSROH) [55], which was suggested to be an appropriate tool for recapitulating oral health status, especially in a low‐resource environment and can be used both in population‐based surveys, as well as clinically to assess patients' oral health needs and treatment progress [56]. More importantly, this tool has been commonly used to assess the impact of oral diseases, for example, an extensive systematic review by van de Rijt et al. [57] concluded that oral health‐related quality of health was inversely associated with retained roots and extensive dental caries, pocket depth, tooth mobility, gingivitis, periodontitis, and abnormalities of the oral mucosa (mouth ulcers, fistulas, and abscesses). Second, the reliance on self‐reported data for dental attendance may introduce recall bias and response bias. This might result in a degree of inaccuracy in exposure measurements. Third, the dichotomization of the outcome variable may oversimplify the nuanced variations within the categories. To address this, additional analyses were conducted with the variable categorized into three groups: self‐rated oral health (excellent, good, and poor). Despite the more detailed categorization, the overall results remained unchanged. These findings are not shown here but are available upon request.

### 4.1 Future Research Recommendations

Future research should aim to explore the pathways through which early dental care behaviors determine long‐term oral health outcomes and identify effective strategies to overcome barriers to dental attendance. Additionally, longitudinal studies incorporating objective clinical assessments of oral health alongside self‐reported measures could enhance the validity of the findings. Efforts to reduce missing data through more rigorous data collection protocols and advanced statistical techniques are also critical in managing biases and producing highly robust results.

## 5 Conclusion

This study demonstrates that childhood dental attendance patterns are positively associated with perceived oral health in adulthood among the UK population, though the strength of this association diminishes after adjusting for covariates. However, the association remains significant among individuals with lower parental academic qualifications and those who brushed their teeth less frequently during childhood. These findings suggest that while regular, early dental visits may contribute to better adult oral health, broader social and behavioral determinants also play a crucial role. This emphasizes the need for comprehensive public health strategies that promote preventive practices, which could reduce oral health inequalities and mitigate the long‐term burden of oral diseases on individuals and healthcare systems.

## Ethics Statement

This study analyzed data from the BCS70 in accordance with the ethical approvals for the original study. Ethics approval for each wave of data collection in the BCS70 was obtained from the National Health Service Research Ethics Committee. Specifically, the Age 46 Survey received approval from the Brighton and Sussex Research Ethics Committee (15/LO/1446).

## Conflicts of Interest

The authors declare no conflicts of interest.

## Supporting information


**Data S1.** Supporting Information.

## Data Availability

The BCS70 data are accessible from the UK Data Service upon registration at https://ukdataservice.ac.uk/.
