# Supplementary material for: Long‐Term Impact of Childhood Dental Attendance on Perceived Adult Oral Health: The British Cohort Study
Source: J Public Health Dent. 2025 Jan 11;85(2):133–42. doi: 10.1111/jphd.12661 (PMC12147437; doi:10.1111/jphd.12661)
Supplement: Supplementary file 1 — Data S1. Supporting Information. [file JPHD-85-133-s001.docx]

**Additional file 1: Multicollinearity test and sensitivity analysis**

**Table S1.** Multicollinearity test to assess the extent of collinearity between predictors (n=4699).

| Variable | VIF | 1/VIF |
| --- | --- | --- |
| Dental attendance pattern |  |  |
| Sometimes regular | 1.06 | 0.95 |
| Never regular | 1.06 | 0.95 |
| Sex | 1.05 | 0.95 |
| Parent’s social class |  |  |
| Skilled non-manual/manual | 1.34 | 0.75 |
| Partially skilled/unskilled | 1.35 | 0.74 |
| Childhood comorbidities | 1.01 | 0.99 |
| Childhood sugary food intake | 1.01 | 0.99 |
| Childhood toothbrushing frequency |  |  |
| < twice daily | 1.23 | 0.81 |
| Unknown | 1.24 | 0.81 |
| Adulthood social class |  |  |
| Intermediate | 1.14 | 0.88 |
| Routine/manual | 1.18 | 0.85 |
| Smoking | 1.03 | 0.97 |
| Adulthood comorbidities | 1.01 | 0.99 |
| Mean VIF | 1.13 |  |

*VIF: variance inflation factor*

**Table S2.** Multivariate logistic regression for self-rated oral health at age 46 years (sensitivity analysis).

|  | **Crude model** | | **Adjusted model** | |
| --- | --- | --- | --- | --- |
|  | **OR [95% CI]** | ***p-*value** | **OR [95% CI]** | ***p-*value** |
|  | n = 4699 |  | n = 2221 |  |
| **Childhood dental attendance pattern** |  |  |  |  |
| Always regular | (Reference) |  | (Reference) |  |
| Sometimes regular | **1.26 [1.09; 1.46]** | **0.002** | 1.00 [0.84; 1.20] | 0.962 |
| Never regular | **1.55 [1.16; 2.06]** | **0.003** | 1.09 [0.77; 1.55] | 0.623 |

*OR: odds ratio; CI: confidence interval*
